# Antioxidant Enzyme Activity and mRNA Expression in the Reproductive Tissues of Male European Red Deer (*Cervus elaphus elaphus*)

**DOI:** 10.3390/ijms26157221

**Published:** 2025-07-25

**Authors:** Nicoletta M. Neuman, Przemysław Gilun, Magdalena Koziorowska-Gilun, Paweł Janiszewski, Anna Dziekońska

**Affiliations:** 1 Department of Animal Biochemistry and Biotechnology, University of Warmia and Mazury in Olsztyn, Oczapowskiego 5, 10-719 Olsztyn, Poland; nicoletta.neuman@uwm.edu.pl (N.M.N.); magda.koziorowska@uwm.edu.pl (M.K.-G.); 2 Institute of Animal Reproduction and Food Research, Polish Academy of Sciences, Trylińskiego 18, 10-683 Olsztyn, Poland; 3 Department of Fur-Bearing Animal Breeding and Game Management, University of Warmia and Mazury in Olsztyn, Oczapowskiego 5, 10-719 Olsztyn, Poland; janisz@uwm.edu.pl

**Keywords:** European red deer, tissues, male reproductive system, antioxidant, genes, protein

## Abstract

The aim of this study was to analyze the influence of season (rut and non-rut) on the antioxidant status of selected reproductive tissues in male European red deer (*Cervus elaphus elaphus*). Tissue samples were collected post mortem from the testes and epididymides (caput, corpus, and cauda) of 24 animals. The activity of antioxidant enzymes (superoxide dismutase—SOD, glutathione peroxidase—GPx, and catalase—CAT) and the mRNA expression of SOD1, SOD2, SOD3, GPx4, GPx5, and CAT were examined. In addition, these proteins were identified by western blot. ANOVA revealed that season, type of tissue, and the interaction between these factors significantly (*p* ≤ 0.05) influenced the activity and mRNA expression of the analyzed enzymes. The activity of SOD and GPx peaked in the corpus epididymis in the rut season and in the caput epididymis in the non-rut season. Regardless of season, the relative abundances of GPx4, SOD1, SOD2, and SOD3 mRNA were highest in the testis, and GPx5 mRNA—in the caput epididymis. The activity of SOD and CAT was significantly higher during the non-rut season compared with the rut season, but only in the caput epididymis. This study demonstrated that the activity of antioxidant enzymes and the relative mRNA expression varies across tissues and seasons to provide the reproductive system of European red deer with the required antioxidant protection. Further research is needed to expand our understanding of the antioxidant defense system in the reproductive tract of European red deer.

## 1. Introduction

Reactive oxygen species (ROS) are produced in all living organisms and play an important role in normal physiological processes [1,2]. However, excessive generation of ROS leads to oxidative stress and has a negative impact on the production and maturation of gametes and all bodily tissues [1,3]. All living organisms possess defense mechanisms against the adverse effects of oxidative stress. The antioxidant defense mechanism relies on the coordinated action of superoxide dismutase (SOD), glutathione peroxidase (GPx), and catalase (CAT), known as the catalytic triad [4,5,6], as well as low-molecular-weight antioxidants such as vitamin C, L-glutathione, and vitamin E [2,7,8].

Superoxide dismutase exists in three isoforms: copper/zinc SOD (Cu/Zn SOD, SOD1), manganese SOD (Mn-SOD, SOD2), and extracellular SOD (Ec-SOD, SOD3) [5]. The SOD1 isoform is localized in the cytoplasm, mitochondrial intermembrane space, and the cell nucleus [9]. The SOD2 isoform has been identified in the mitochondrial matrix [10], and SOD3—in the testes and epididymides of various animals, including the European bison [6]. In the disproportionation reaction, SOD neutralizes the superoxide radical (O_2_^•−^) to form toxic hydrogen peroxide (H_2_O_2_), which is then broken down by CAT or GPx [4,11,12]. Catalase shows affinity for H_2_O_2_ when this compound occurs at a high concentration in a cell. However, H_2_O_2_ is degraded by GPx when its levels are low [13]. Cytosolic glutathione peroxidase (GSH-Px) protects testes against toxic H_2_O_2_ [14]. Two isoforms of GPx—phospholipid hydroperoxide glutathione peroxidase (PHGPx/GPx4) and GPx5—have been identified in the reproductive system of many mammalian species [2,6,15,16,17]. The GPx4 isoform is localized in the testes and spermatozoa, whereas GPx5 is present in the plasma membrane of male gametes and in the caput epididymis [18]. In mammals, GPx4 and GPx5 play a key role in the antioxidant defense system of the male reproductive system [2,18,19], and they participate in fertilization processes [20,21,22].

The red deer (including European red deer) is a wild animal with considerable ecological and economic significance [23,24]. Due to its feeding habits, this species plays an important role in the forest ecosystem, influencing the diversity and structure of vegetation in the environment. Furthermore, it provides a food source for predators such as wolves and lynxes, which influences the balance of the entire ecosystem. Red deer reproduction is characterized by synchronized timing of breeding and calving, which is crucial for population development and is dependent on environmental factors, including food availability and the climate [25,26]. Understanding the ecological aspects and physiology of wild animals, including the European red deer, can contribute to the development of an effective conservation strategy for various cervid species and better management of their populations.

The rut season of the European red deer typically occurs in September and October [27]. During the rut season, hormonal changes initiate spermatogenesis and can significantly affect oxidative stress levels in reproductive tissues [28]. Previous research has shown that oxidative stress has a negative impact on semen quality and the fertilizing capacity of spermatozoa [8,17]. Most studies on oxidative stress and antioxidant defenses in the male reproductive system have been conducted on livestock and companion animals [12,16,28,29,30,31,32], whereas the number of scientific reports concerning free-living animals is limited [6,15,33]. There is a general scarcity of research on the antioxidant status of the reproductive system of European red deer stags during the rut and non-rut seasons. Therefore, the present study was conducted on the assumption that season (rut vs. non-rut) can significantly affect the antioxidant status of reproductive tissues in European red deer.

In view of the above, the aim of this study was to analyze the influence of season (rut season and non-rut season) on the antioxidant status of selected reproductive tissues in male European red deer (*Cervus elaphus elaphus*). This goal was achieved by analyzing the activity of antioxidant enzymes (SOD, GPx, and CAT) and the relative abundance of mRNA of SOD1, SOD2, SOD3, GPx4, GPx5, and CAT genes. The presence of these proteins, i.e., SOD1, SOD2, SOD3, GPx4, GPx5, and CAT, in the testes and epididymides (caput, corpus, and cauda) was also determined by western blot.

## 2. Results

The ANOVA revealed that season (rut and non-rut), tissues (testis and caput, corpus, and cauda epididymis), and the interaction between these factors significantly (*p* ≤ 0.05) influenced all activities of the antioxidant enzymes SOD, GPx, and CAT (Table 1).

The sampling season significantly (*p* < 0.05) affected the expression of SOD3, GPx4, GPx5, and CAT genes (Table 2). Type of tissue highly significantly (*p* < 0.001) affected the mRNA expression of the examined genes. In turn, the season × tissue interaction significantly influenced the mRNA expression of all genes, excluding the SOD3 gene.

### 2.1. Activity of Antioxidant Enzymes

**Figure 1:**
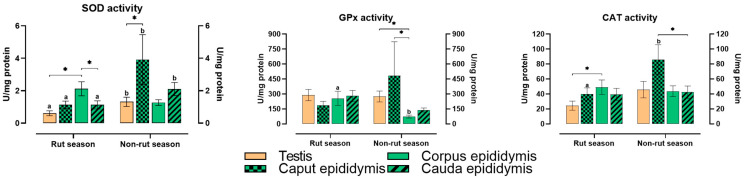
Activity of antioxidant enzymes in the reproductive tissues of male European red deer in rut and non-rut seasons. SOD activity—superoxide dismutase activity; GPx activity—glutathione peroxidase activity; CAT activity—catalase activity. The mean (±SEM) values of antioxidant enzyme activity are presented. a, b—significant at *p* ≤ 0.05 between seasons. Values marked with * denote significant differences at *p* ≤ 0.05 between the analyzed tissues in each season.

The sampling season (rut and non-rut) and type of tissue significantly influenced the activity of antioxidant enzymes (Figure 1). The activity of SOD in the testis and in the caput and cauda epididymis was significantly (*p* ≤ 0.05) higher during the non-rut season compared with that during the rut season. During the rut season, this parameter was also significantly higher in the corpus epididymis than in the testis and cauda epididymis. During the non-rut season, SOD activity was significantly higher in the caput epididymis than in the testis.

Significant seasonal differences in GPx activity were noted only in the corpus epididymis, where the examined parameter was significantly higher during the rut season. However, GPx activity peaked in the caput epididymis during the non-rut season. During the non-rut season, significant differences in GPx activity were observed between the corpus epididymis vs. the testis and the caput epididymis.

Season affected CAT activity only in the caput epididymis. In this tissue, CAT activity was significantly higher in the non-rut season compared with that in the rut season. During the rut season, CAT activity was significantly higher in the corpus epididymis than in the testis. During the non-rut season, CAT activity was significantly higher in the caput epididymis than in the cauda epididymis.

### 2.2. Relative Abundance of Analyzed Enzymes

**Figure 2:**
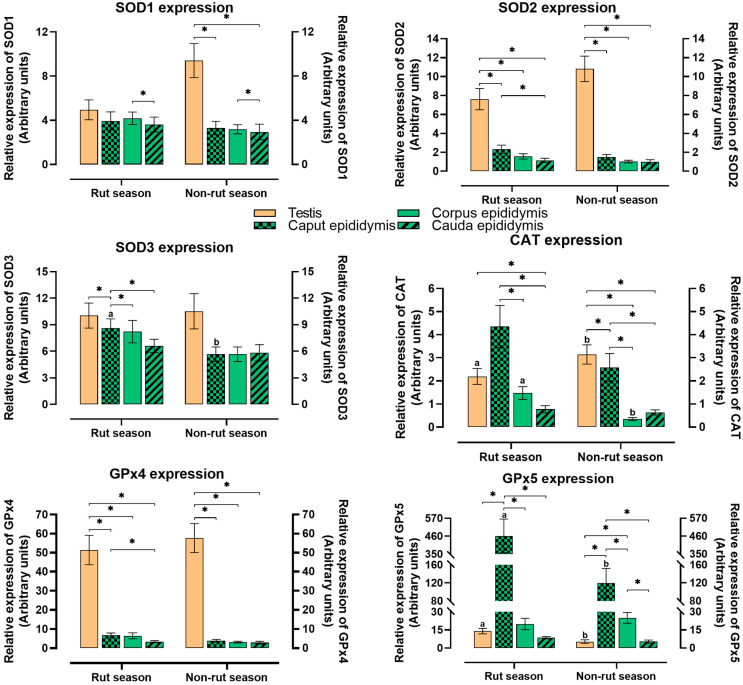
Relative mRNA abundance of the analyzed genes in the reproductive tissues of male European red deer in rut and non-rut seasons. SOD1 expression—relative abundance of cytoplasmic copper/zinc superoxide dismutase mRNA; SOD2 expression—relative abundance of manganese superoxide dismutase mRNA; SOD3 expression—relative abundance of extracellular superoxide dismutase mRNA; GPx4 expression—relative abundance of glutathione peroxidase 4 mRNA; GPx5 expression—relative abundance of glutathione peroxidase 5 mRNA; CAT expression—relative abundance of catalase mRNA. The mean (±SEM) values of relative mRNA abundance are presented. a, b—significant at *p* ≤ 0.05 between seasons. Values marked with * denote significant differences at *p* ≤ 0.05 between the analyzed tissues in each season.

The abundance of SOD1 mRNA in the examined tissues did not differ significantly between seasons. Regardless of season, the relative abundance of SOD1 mRNA was significantly (*p* ≤ 0.05) higher in the corpus epididymis than in the cauda epididymis (Figure 2). During the non-rut season, the examined parameter was significantly higher in the testis than in the caput and cauda epididymis.

Regardless of season, the relative abundance of SOD2 mRNA was significantly (*p* ≤ 0.05) higher in the testis than in the epididymis (caput, corpus, and cauda). During the rut season, the relative abundance of SOD2 mRNA was significantly higher in the caput epididymis than in the cauda epididymis.

Seasonal differences in the abundance of SOD3 mRNA were observed only in the caput epididymis. In this tissue, the relative abundance of SOD3 mRNA was significantly (*p* ≤ 0.05) higher in the rut season than in the non-rut season. In both seasons, the relative abundance of SOD3 mRNA was highest in the testis, and in the rut season, significant differences in this parameter were noted between the testis and the caput epididymis, and between the caput epididymis and the corpus and cauda epididymis.

Significant seasonal variations in the relative abundance of CAT mRNA were observed in the testis and the corpus epididymis. During the rut season, the relative abundance of CAT mRNA was significantly (*p* ≤ 0.05) lower in the testis and higher in the corpus epididymis than during the non-rut season. Regardless of the season, the analyzed parameter was significantly higher in the caput epididymis than in the corpus and cauda epididymis, and significantly higher in the testis than in the cauda epididymis. During the non-rut season, the relative abundance of CAT mRNA was significantly higher in the testis than in the caput epididymis.

Regardless of season, the relative abundance of GPx4 mRNA was significantly (*p* ≤ 0.05) higher in the testis than in the epididymis. During the rut season, this parameter was significantly higher in the caput epididymis than in the cauda epididymis.

Significant seasonal differences in the relative abundance of GPx5 mRNA were observed in the testis and the caput epididymis. In these tissues, the analyzed parameter was significantly (*p* ≤ 0.05) higher in the rut season than in the non-rut season. Regardless of season, the relative abundance of GPx5 mRNA was significantly highest in the caput epididymis. During the non-rut season, the relative abundance of GPx5 mRNA was significantly higher in the corpus epididymis than in the testis and the cauda epididymis.

### 2.3. Western Blot Analysis

**Figure 3:**
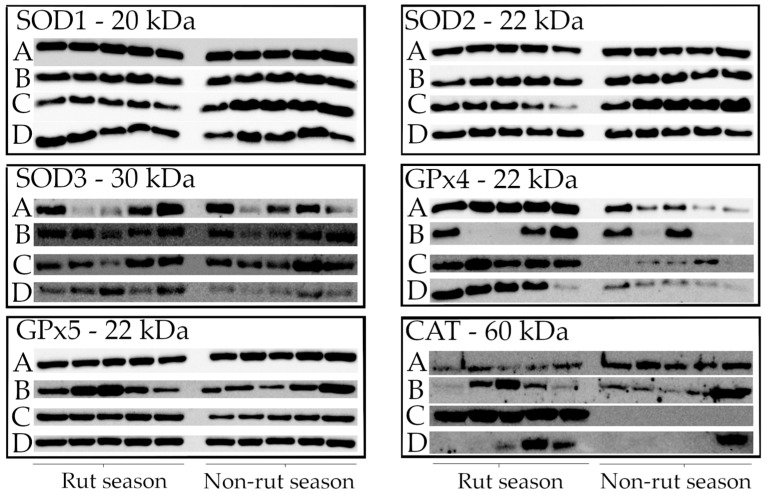
Western blot analysis of SOD1—cytoplasmic copper/zinc superoxide dismutase; SOD2—manganese superoxide dismutase; SOD3—extracellular superoxide dismutase; GPx4—glutathione peroxidase 4; GPx5—glutathione peroxidase 5; CAT—catalase in the testis (A), caput epididymis (B), corpus epididymis (C), and cauda epididymis (D) in rut (*n* = 5) and non-rut (*n* = 5) seasons.

The western blot technique was used to determine the protein content of tissues sampled from randomly selected animals (*n* = 5 in the rut season; *n* = 5 in the non-rut season) (Figure 3). Tissue samples were loaded onto gels in the same sequence. All analyzed proteins were identified. SOD1, SOD2, and SOD3 proteins were detected in all animals, which corroborates the results of gene expression and enzyme activity analyses. The results of GPx4, GPx5, and CAT protein analyses were also consistent with the gene expression data.

## 3. Discussion

To the best of the authors’ knowledge, this is the first study to investigate the influence of rut and non-rut seasons on the antioxidant status of selected reproductive tissues in male European red deer (*Cervus elaphus elaphus*). Previous studies analyzing reproductive tract tissues were conducted on roe deer, European bison, horses, and wild boar/domestic pig hybrids [6,15,16,30]. The antioxidant status of human reproductive tissues has been investigated most extensively [34,35,36].

The antioxidant system prevents ROS accumulation in cells because excessive ROS production has an adverse effect on cells and leads to oxidative stress [12,31]. In all living organisms, ROS are generated during basic cellular processes, including oxidative phosphorylation in the electron transport chain [3], spermatogenesis, and sperm maturation in the epididymides [31,37]. Small amounts of ROS play important roles in the body by inducing apoptosis, stimulating glucose transport to cells, and participating in sperm hyperactivation, capacitation, and acrosome reactions [3,12,14,31]. In turn, oxidative stress can lead to protein oxidation and damage to nucleic acids, membrane phospholipids, and chromosomes [3,8,32].

In many mammalian species, the antioxidant system is composed of enzymatic and non-enzymatic antioxidants [38]. Superoxide dismutase is the key antioxidant enzyme in animals [39]. The present study revealed considerable differences in SOD activity in the testes and epididymides, and in the mRNA expression of the examined SOD isoforms between the rut season and the non-rut season. Regardless of season, substantial differences were also observed between the analyzed tissues. In the epididymis, SOD3 mRNA was more highly expressed than SOD1 and SOD2 mRNA. The expression of SOD2 and SOD3 mRNA was higher in the testis than in the epididymis. These results could indicate that SOD2 and SOD3 play a more important role in the antioxidant defense of the testes than SOD1. Similar observations were made in the reproductive tissues of European bison [6]. However, all three SOD isoforms are required for effective protection against the harmful effects of ROS [5]. In the current study, the expression of SOD2 mRNA was highest in the testis, regardless of season. Similar results were reported in a study of mice [40]. The functions of all SOD isoforms have not yet been fully explored. However, research has shown that SOD3 protects the testes and spermatozoa against lipid peroxidation. Lipid peroxidation damages sperm DNA, decreases sperm motility, and alters cell membrane permeability, thus compromising the fertilizing capacity of spermatozoa [8,14,41]. Sperm cells are particularly susceptible to lipid peroxidation because their membranes are abundant in polyunsaturated fatty acids (PUFAs) [14,42]. Peltola et al. [43] demonstrated that ROS are produced by Leydig cells and Sertoli cells in the testes. In addition, large amounts of ROS are generated during spermatogenesis [10]. In this case, SOD2 and SOD3 probably play a key role in enzymatic reactions that prevent ROS overproduction in the testes [6].

In the present study, similar changes in CAT and SOD activity were observed in the examined reproductive tissues. This dependency confirms that CAT breaks down H_2_O_2_ that is produced by SOD in cells [10,12]. Regardless of season, CAT activity was higher in the epididymides than in the testes, which could suggest that H_2_O_2_ produced during sperm maturation is more rapidly degraded in the epididymides than in the testes [15]. In European red deer, the expression of CAT mRNA was highest in the caput epididymis during the rut season. Similar results were reported for European bison and roe deer [6,15], as well as for rats [29]. The expression of the CAT gene was low in the testis during the rut season, which corroborates the findings of Zini and Schlegel [29], who argued that the testes cannot directly remove H_2_O_2_. In this study, the expression of CAT mRNA was not correlated with CAT activity, which was also observed in the authors’ previous research [6,15]. This fact can be attributed to the mechanism that regulates gene expression. Reactive oxygen species control transcription factors that regulate CAT mRNA expression and the translation of the CAT gene [44,45]. In European red deer, this mechanism could have affected the regulation of CAT mRNA expression in the testis and in the caput epididymis. Catalase was probably the primary antioxidant enzyme in these reproductive tissues.

Glutathione peroxidase also breaks down H_2_O_2_ in cells, but it relies on a different mechanism of action than CAT [17,18,46]. Catalase is activated in response to a high concentration of H_2_O_2_. Research has shown that some enzymes, including antioxidant enzymes, can be activated by oxidative bursts that may occur in the body during oxidative stress [2,17].

In this study, GPx activity peaked in the testes during the rut season, and the expression of GPx4 mRNA was higher in the testes than in the other tissues, regardless of season. Similar relationships were noted by Vernet et al. [17], Foresta et al. [47], and Koziorowska-Gilun et al. [6,15]. This observation confirms that high GPx activity can compensate for low CAT activity in the testes. In the present study, the abundance of GPx5 mRNA was much lower in the testes than in the epididymides, regardless of season. This gene was most highly expressed in the caput epididymis. Similar results were reported in studies of the male reproductive system in rats, bulls, mice, sheep, and pigs [21,32,48,49,50]. The expression of GPx5 mRNA was lower in the cauda epididymis than in the corpus epididymis. This finding corroborates previous observations made in sheep [32]. Glutathione peroxidase 5 is found specifically in the epididymides, and it has been identified in different segments of the epididymis in various animal species [2,18,21]. According to many researchers, seasonal changes in hormone levels influence the reproductive system in numerous animal species [51,52]. In Cerviade during the rutting period, the maximal amount of testosterone in serum and seminal plasma, large size, and intensive meiotic activity of testes have been observed by Lincoln [53], Barrell et al. [54], Roelants et al. [55], Goeritz et al. [56], and Kozioł and Koziorowski [57]. In addition, Aitken and Roman [5] found that seasonal fluctuations in hormone levels in the testes and epididymides significantly affect the activity and expression of antioxidant enzymes. Marchlewicz et al. [31] and Belleannèe et al. [58] posited that androgens indirectly regulate GPx5 expression through a tissue-specific transcription factor. In goats, testosterone enhances GPx5 expression through androgen receptors [59]. Cervids are seasonal breeders, and hormonal changes could have also influenced the expression of the GPx gene. In the current study, hormonal fluctuations could have also been responsible for significantly higher GPx5 expression in the testis and the caput epididymis during the rut season compared with that in the non-rut season. However, further research involving analyses of hormone levels is required to confirm these observations.

## 4. Materials and Methods

### 4.1. Animals and Sample Collection

The testes and epididymides were obtained from 24 European red deer (*Cervus elaphus elaphus*) stags that were culled during legal hunts in Nowe Ramuki Forest District (Region of Warmia and Mazury, Poland). Tissue samples for analysis were collected during the rut season (September–October, *n* = 12) and the non-rut season (December–February, *n* = 12). The samples were collected within five hours post mortem and were transported to the laboratory of the Department of Animal Biochemistry and Biotechnology of the University of Warmia and Mazury in Olsztyn. Tissue samples were obtained from different parts of the reproductive tract, i.e., the testis and the caput, corpus, and cauda epididymis. For each tissue and individual, a comparable amount of material—approximately 1 cm^3^—was collected, regardless of season. The collected tissues were frozen in liquid nitrogen and stored at a temperature of −80 °C until further analysis. Tissue samples for gene expression analyses were preserved in TRI-Reagent solution.

### 4.2. Tissue Preparation

#### 4.2.1. Antioxidant Enzyme Activity

The collected samples were homogenized in a tissue extraction buffer (50 mM Tris, 5 mM ethylenediaminetetraacetic acid—EDTA, 1 mM dithiothreitol—DDT, 1 μg/mL aprotinin, pH 7.5) in Lysing Matrix Tubes (MP Biomedicals LLC, Solon, OH, USA) in a FastPrep-24 apparatus (MP Biomedicals LLC, Solon, OH, USA) for 45 s to determine the activity of antioxidant enzymes. The homogenates were centrifuged at 15,000× *g* and a temperature of 4 °C for 15 min, and total protein content was determined in the Bradford assay [60]. The prepared samples were used to determine the activity of antioxidant enzymes.

#### 4.2.2. Real-Time PCR

The tissues of the testis and epididymis (caput, corpus, and cauda) were homogenized in Lysing Matrix Tubes (MP Biomedicals LLC, Solon, OH, USA) containing 1 mL of TRI-Reagent in a FastPrep-24 apparatus (MP Biomedicals LLC, Solon, OH, USA) for 45 s. RNA was isolated from the homogenized samples with a Total RNA Kit (A&A Biotechnology, Gdynia, Poland) according to the manufacturer’s protocol. RNA quality was assessed based on the 260:280 absorbance ratio.

#### 4.2.3. Western Blot

Protein was extracted from tissues with TKM buffer (50 mM Tris-HCl, pH 7,4; 25 mM KCl; 5 mM MgCl_2_; 1 mM EDTA). Tissues were ground in liquid nitrogen using a pre-chilled pestle and mortar, suspended in TKM buffer, and homogenized for 45 s. In the following step, the homogenized samples were combined with TKM buffer (1% Triton X-100) and centrifuged at 8000× *g* at a temperature of 4 °C for 10 min. The supernatants were stored at −20 °C. The total protein content of each sample was determined in the Bradford assay [60].

### 4.3. Sample Analyses

#### 4.3.1. Antioxidant Enzyme Activity

The activity of the analyzed antioxidant enzymes was measured with a Beckman Coulter DU 800 spectrophotometer (Beckman Coulter Inc., Fullerton, CA, USA) and expressed in U/mg of protein.

- Superoxide dismutase activity

The activity of SOD was determined with a Ransod kit (Randox Laboratories, Crumlin, UK), and the samples were prepared based on the manufacturer’s instructions. Superoxide dismutase activity was measured at a wavelength of 505 nm.

- Glutathione peroxidase activity

The activity of GPx was determined with a Ransel kit (Randox Laboratories, Crumlin, UK), and the samples were prepared according to the manufacturer’s instructions. Glutathione peroxidase activity was measured at a wavelength of 340 nm.

- Catalase activity

Catalase activity was analyzed with a Catalase Assay Kit (Sigma-Aldrich Co., Saint Louis, MO, USA) according to the manufacturer’s instructions. Catalase activity was determined at a wavelength of 520 nm by measuring the amount of H_2_O_2_ remaining after the reaction catalyzed by CAT.

#### 4.3.2. Gene Expression

##### Reverse Transcription Polymerase Chain Reaction

The reverse transcription polymerase chain reaction (RT-PCR) was performed with the RevertAid™ Premium First Strand cDNA Synthesis Kit (Thermo Fischer Scientific Inc., Waltham, MA, USA) based on the manufacturer’s instructions. In brief, 1 µg of RNA from each tissue sample was used in RT-PCR, and cDNA was synthesized in a PCR Thermal Cycler (MJ Mini Thermal Cycler, Bio-Rad Laboratories Inc., Hercules, CA, USA). The assay consisted of the following temperature cycles: 25 °C for 10 min, 42 °C for 60 min, and 70 °C for 5 min, followed by 4 °C. Single-stranded DNA was diluted with nuclease-free water (1:9) and used in real-time PCR.

##### Real-Time PCR

The obtained cDNA was used for the qPCR assay in the Real-Time PCR ABI 7900HT system (Applied Biosystems, Foster City, CA, USA) according to a previously described method [6] with some modifications. The reaction mix was composed of 2 µL of the cDNA matrix, 5 µL of each primer (forward and reverse), 12.5 µL of the Maxima SYBR/ROX PCR Mix (Thermo Fischer Scientific Inc., Waltham, MA, USA), and 5.5 µL of nuclease-free water. Hot Start DNA polymerase was activated at 95 °C for 10 min. The PCR assay consisted of 40 cycles, including denaturation at 95 °C for 10 min, followed by primer annealing and elongation at 60 °C for 60 s. Each sample was assayed in duplicate. The primers were designed using Primer Express Software v 3.0 (Applied Biosystems, Foster City, CA, USA) based on the red deer genome (Table 3). The expression of the analyzed genes (SOD1, SOD2, SOD3, GPx4, GPx5, and CAT) was quantified by comparing their relative mRNA abundance with that of the reference gene (SDHA), which was selected based on its superior stability in the analyzed tissues compared with other tested reference genes, including β-actin (ACTB), GAPDH, and 18S rRNA. The expression of the analyzed genes (SOD1, SOD2, SOD3, GPx4, GPx5, and CAT) was quantified by comparing their relative mRNA abundance with that of the reference gene (SDHA). The absolute quantification of mRNA was performed using the Real Time PCR Miner algorithm [61].

#### 4.3.3. Western Blot

##### SDS-PAGE

Electrophoresis was conducted on a 15% polyacrylamide gel with SDS [62] in a Mini-Protean II Cell (Bio-Rad Laboratories, Hercules, CA, USA) using a Tris-Glycine buffer (25 mM Tris, 250 mM glycine, 0.1% SDS, pH 8.3). Each sample contained 20 µg of protein and 2 µL of a loading buffer (1 M Tris-HCl, 20% SDS, 20% glycerol, 2% β-mercaptoethanol, 2% bromophenol blue, pH 6.8). The prepared samples were loaded into wells.

##### Semi-Dry Protein Transfer

The separated proteins were transferred to an Immobilon-P polyvinylidene fluoride (PVDF) membrane in the Pierce Power Blot system (Thermo Fisher Scientific, Waltham, MA, USA). The membrane was blocked overnight with 5% skim milk powder in TBS-T buffer (1 M Tris, 5 M NaCl, 0.05% (*v*/*v*) Tween 20) at a temperature of 4 °C.

##### Antibody Incubation

The analyzed proteins were identified using the following primary antibodies:

- Rabbit polyclonal anti-SOD1 antibody (1:10,000; PA5-23245; Thermo Fisher Scientific, Waltham, MA, USA);
- Rabbit anti-SOD2 polyclonal antibody (1:1000; PA1-31072; Thermo Fisher Scientific, Waltham, MA, USA);
- Rabbit polyclonal anti-SOD3 antibody (1:1000; PA5-93329; Thermo Fisher Scientific, Waltham, MA, USA);
- Rabbit polyclonal anti-GPx4 antibody (1:1000; ab41787; Abcam, Cambridge, UK);
- Rabbit polyclonal anti-GPx5 antibody (1:500; PA5-102342; Thermo Fisher Scientific, Waltham, MA, USA);
- Rabbit polyclonal anti-CAT antibody (1:15,000; 200-401-051; Thermo Fisher Scientific, Waltham, MA, USA);
- Mouse monoclonal anti-beta actin (C4) antibody (1:200; sc-47778; Santa Cruz Biotechnology, Dallas, TX, USA).

The membranes were incubated overnight with primary antibodies at a temperature of 4 °C. After incubation, the membranes were rinsed and incubated with goat anti-rabbit IgG secondary antibodies conjugated to HRP (1:15,000; sc-2004; Santa Cruz Biotechnology, Dallas, TX, USA) for 1 h at room temperature. Protein bands were visualized by chemiluminescent detection in the ChemiDoc imaging system (Bio-Rad Laboratories, Hercules, CA, USA) with the SuperSignal^®^ West Dura Extended Duration Substrate.

### 4.4. Statistical Analysis

The results were processed statistically in the Statistica v. 14.1.0.4 program (StatSoft Inc., Tulsa, OK, USA). The normality of data distribution was checked via the Shapiro–Wilk test. Non-normally distributed data were transformed with the Box–Cox transformation. The effect of season on the studied parameters was compared by one-way analysis of variance (ANOVA). The significance level (*p* ≤ 0.05) was adjusted via the Bonferroni post hoc test. The results were expressed as mean values ± SEM.

## 5. Conclusions

In summary, the present study revealed considerable differences in the activity and mRNA expression of antioxidant enzymes in the reproductive tissues of male European red deer between the rut season (September-October) and the non-rut season (December-February). In addition, this study demonstrated that the key antioxidant enzymes—SOD, GPx, and CAT—participated in the antioxidant defense system of reproductive tissues in European red deer stags in both examined periods, particularly during the rut season. Considering the specific hormonal regulation in cervids, characterized by a surge in testosterone levels during the rutting season, it can be assumed that the observed changes in the activity as well as mRNA and protein levels of the studied antioxidant enzymes are related to fluctuations in sex hormone concentrations between the rutting and non-rutting periods. However, further research is needed to expand our understanding of the antioxidant defense system in the reproductive tract of European red deer.

## Figures and Tables

**Table 1:** The effect of season and tissue on enzyme activity in ANOVA.

Enzyme Activity	Season		Tissue		Season × Tissue	
	*F* Value	*p* Value	*F* Value	*p* Value	*F* Value	*p* Value
SOD activity	19.968	<0.001	10.323	<0.001	7.780	<0.001
GPx activity	7.437	0.007	7.868	<0.001	4.285	0.006
CAT activity	7.803	0.006	5.940	<0.001	3.160	0.026

Values are significant at *p* ≤ 0.05; SOD activity—superoxide dismutase activity; GPx activity—glutathione peroxidase activity; CAT activity—catalase activity.

**Table 2:** The effect of season and tissue on gene expression in ANOVA.

Gene Expression	Season		Tissue		Season × Tissue	
	*F* Value	*p* Value	*F* Value	*p* Value	*F* Value	*p* Value
SOD1 expression	0.610	n.s.	11.576	<0.001	3.330	0.020
SOD2 expression	1.280	n.s.	137.558	<0.001	4.700	0.003
SOD3 expression	16.495	<0.001	12.885	<0.001	2.455	n.s.
GPx4 expression	6.442	0.012	254.887	<0.001	2.712	0.045
GPx5 expression	34.054	<0.001	100.495	<0.001	6.300	<0.001
CAT expression	5.639	0.018	53.686	<0.001	12.883	<0.001

Values are significant at *p* ≤ 0.05; n.s.—not significant; SOD1 expression—relative abundance of cytoplasmic copper/zinc superoxide dismutase mRNA; SOD2 expression—relative abundance of manganese superoxide dismutase mRNA; SOD3 expression—relative abundance of extracellular SOD mRNA; GPx4 expression—relative abundance of glutathione peroxidase 4 mRNA; GPx5 expression—relative abundance of glutathione peroxidase 5 mRNA; CAT expression—relative abundance of catalase mRNA.

**Table 3:** Sequences of synthetic primers applied in real-time reverse-transcription polymerase chain reaction (RT-PCR) to quantify gene expression in the reproductive tissues of male European red deer. Each primer set was designed based on the accession number in the European Molecular Biology Laboratory database.

Gene	Forward Primer 5′–3′	Reverse Primer 5′–3′	Accession Number
SDHA	GACAGGAGCCCGCAGTTTT	CACGGCATCAAACTCATGGT	XM_043887904.1
SOD1	CTCCTTTTCCCCGAGTCATG	CGACGACTGTATTTCCCTTTGC	XM_043892610.1
SOD2	TCTGCAGCCTGCGTTAAAGTT	CTTCCAGCAATTCCCCTTTG	XM_043888243.1
SOD3	CGCTGCTCTGTGCCTATCTG	TGTGCATGTCGCGGATCT	XM_043870913.1
GPx4	TACGCCGAGTGTGGTTTACG	GCGGCGAACTCTTTGATCTC	XM_043912658.1
GPx5	TGACATCCGCTGGAATTTTG	ACTGACGGGAGTCCGATGAA	XM_043908264.1
CAT	TGCCCTATTGTTTCCGTCCTT	ACCATGTCCGGATCCTTCAG	XM_043909065.1

SDHA—reference gene, succinate dehydrogenase complex flavoprotein subunit A; SOD1—cytoplasmic copper/zinc superoxide dismutase; SOD2—manganese superoxide dismutase; SOD3—extracellular superoxide dismutase; GPx4—glutathione peroxidase 4; GPx5—glutathione peroxidase 5; CAT—catalase.

## Data Availability

The data presented in this study are available on request from the corresponding author.
